# Trends (2007–2019) of major atopic diseases throughout the life span in a large Mexican population

**DOI:** 10.1016/j.waojou.2022.100732

**Published:** 2023-01-09

**Authors:** Martín Becerril-Ángeles, Mario H. Vargas, Ismael Seth Medina-Reyes, Ramón Alberto Rascón-Pacheco

**Affiliations:** a Departamento de Alergia e Inmunología Clínica, Hospital de Especialidades, Centro Médico Nacional La Raza, Instituto Mexicano del Seguro Social, Mexico City, Mexico; b Hospital Médica Sur, Mexico City, Mexico; c Departamento de Investigación en Hiperreactividad Bronquial, Instituto Nacional de Enfermedades Respiratorias Ismael Cosío Villegas, Mexico City, Mexico; d Coordinación de Vigilancia Epidemiológica, Instituto Mexicano del Seguro Social, Mexico City, Mexico

**Keywords:** Asthma, Allergic rhinitis, Atopic dermatitis, Atopic eczema, Atopic disease, AD, atopic dermatitis, AR, allergic rhinitis, IMSS, Instituto Mexicano del Seguro Social, M:F, male:female

## Abstract

**Background:**

Major atopic diseases such as atopic dermatitis (AD), allergic rhinitis (AR), and asthma share the same atopic background, but they often show differences in their epidemiological behavior.

**Objective:**

We aimed to report the profile of these atopic diseases in a large Mexican population, including their age-related incidences, male:female (M:F) ratios, recent time trends, and association with altitude.

**Methods:**

Registries from the largest, nationwide health institution in Mexico (more than 34 million insured subjects), were reviewed. New cases of AD, AR, and asthma diagnosed each year by family physicians from 2007 to 2019 were adjusted by the corresponding insured population to estimate incidence rates.

**Results:**

Incidences of the 3 atopic diseases were highest in the 0–4 years age-group and progressively decreased thereafter until adolescence. Asthma and AR, but not AD, were more frequent in males during childhood (M:F ratios of 1.5, 1.3, and 0.95, respectively), but predominated in females during adulthood (M:F ratios of 0.52, 0.68, and 0.73, respectively). Time trends showed an initial increasing trend of annual incidences, with a peak around 2009–2011, and a downward trend afterward. This decreasing trend was seen in all age-groups and was more evident for AD (∼50% drop) and asthma (∼40% drop) than for AR (∼20% drop). Geographical distribution suggested that incidences of asthma and AR, but not of AD, had an inverse association with altitude.

**Conclusion:**

Annual incidences of the 3 major atopic diseases have declined in recent years in almost all age groups, and their epidemiological profile during the life span showed contrasting differences according to age, sex, and ecological association with altitude, mainly regarding AD.

## Introduction

An epidemic rising trend in the prevalence of atopic diseases was a worldwide phenomenon between 1960s and the late 1990s, but this trend was followed by a plateau in many countries, lacking until now a clear explanation for this behavior.^1^ Atopic diseases comprise 4 major conditions: atopic dermatitis (AD), food allergy, allergic rhinitis (AR), and asthma. A number of factors have been associated with the development of these diseases, including genetic traits,^2^ maternal smoking,^3^ respiratory viral infections,^4^ and obesity.^5^ By contrast, some conditions seem to be protective for the development of atopic diseases as, for example, exposure to bacterial endotoxins from livestock, mainly during early life.^6^ Moreover, the capability of skin, intestinal and/or respiratory microbiota to modulate immune responses and the possible role of its dysbiosis in allergy is gaining interest.^7^, ^8^, ^9^

These allergic diseases are involved in the so-called atopic march. The term "atopic march" was coined to depict the progressive appearance of several allergic diseases during childhood, usually following the sequence of AD, food allergy, AR, and asthma.^10^ The extended “epithelial barrier hypothesis” postulates that even trace amounts of substances linked to industrialization, urbanization, and modern life can damage epithelial barriers and increase bacterial translocation.^11^^,^^12^ In the skin, this anomalous epithelium would favor the inception of AD,^13^ which in turn promotes the development of food and airway allergies through an early systemic sensitization, as has been shown by genetic association studies, cross-sectional studies, cohort studies, and experimental animal models.^14^^,^^15^ The severity of AD in infancy seems to be an important risk factor for respiratory allergy later in life. Thus, infants with early-onset persistent AD had a three-fold risk for asthma and AR in the next few years, as compared with children who developed AD after 2 years of age.^16^ In a Swedish cohort, in children under 3 years of age the inception of asthma by age 7 was different in infants with severe AD and those with mild AD, amounting to >60% and 20%, respectively.^17^

In order to gain insights about the time trends from 2007 to 2019 in annual incidences of AD, AR, and asthma, and how these incidences change according to sex and age-group, databases of the Instituto Mexicano del Seguro Social (IMSS), which is the largest health institution in Mexico, were reviewed. In 2019, its workforce of more than 17 800 family physicians across the country provided medical services to the population of insured subjects formally registered in a first-contact medical unit (non-government employees and their spouses, daughters, sons, and parents). This insured population ranged from ∼34.8 (year 2007) to ∼50.9 (year 2019) million subjects, representing more than one-third of the Mexican population. On the other hand, Mexico has a large diversity of regional conditions, including altitude, and it has been previously described that there is an inverse association between asthma and altitude, starting from 1500 m above sea level.^18^ Thus, an additional objective of the present study was to explore this association and to evaluate whether it is also present in AR and AD.

## Methods

### Study design

Dynamics of the data gathering process at IMSS has been described elsewhere.^18^^,^^19^ Briefly, medical consultations provided and registered by each family physician are concentrated in a nationwide database called SUI27. From this database, medical diagnoses from patients of any age made each year from 2007 to 2019 concerning first-time medical consultations (ie, newly diagnosed cases) were analyzed both in a nationwide context and broken down among the ∼740 counties where the IMSS provides medical services to its ordinary regimen insured population. Although these counties correspond to ∼27% of all Mexican counties, they concentrate ∼79% of the Mexican population. Medical units in these counties also provide medical care to insured persons from the nearest small, low-populated rural counties not having medical units. Atopic diseases included in the study were asthma (ICD-10 codes J45 and J46), AR (ICD-10 code J30), and AD (ICD-10 code L20). According to institutional regulations, for establishing the diagnosis of asthma, AR and/or AD, family physicians must adhere to official and updated clinical practice guidelines,^20^, ^21^, ^22^ which in turn follow international recommendations, thus assuring a reasonable level of homogeneous diagnostic criteria. Moreover, family physicians are specialists with a full three-year residency for training in the diagnosis and treatment of the most frequent diseases occurring in the insured population. Food allergy, anaphylaxis, urticaria, and allergy to drugs were not included in the analysis because these are more complex diseases that often require the diagnostic workup at the secondary or tertiary care level. The counties’ altitudes above sea level were obtained from the publicly available database of the National Institute of Statistics and Geography (INEGI).

Because this was a retrospective analysis of a de-identified institutional database, it was deemed unnecessary obtaining the approval from an ethics and scientific review board.

### Data analysis

Because only newly diagnosed cases were analyzed, when they were divided by the cumulative number of insured subjects of the same sex and in the same location, time-period and age-group, incidence rate was obtained, ie, the number of insured subjects (denominator) exactly matched the characteristics of cases (numerator) regarding sex, age, years analyzed, and geographical limits. Crude and age-adjusted (Poisson regression) male:female (M:F) ratios were calculated using incidence in subjects <15 years old and subjects ≥15 years old. Non-paired Student's t-test was used for group comparisons. Statistical significance was set at two-tailed p < 0.05. Data were processed in Microsoft Excel spreadsheets.

## Results

As can be seen in Table 1, the population studied ranged from ∼34.8 to ∼50.9 million insured subjects, and in the 13 years studied the number of patients diagnosed with any of the 3 atopic diseases (AD, asthma, or AR) totalized 9.7 million (∼744,000 patients per year).

**Table 1:** Nationwide annual incidences of the 3 major atopic diseases by year

Year	Population^a^	Cases			Incidences (x100,000)^b^		
		Atopic dermatitis	Asthma	Allergic rhinitis	Atopic dermatitis	Asthma	Allergic rhinitis
2007	34,753,769	59,706	382,871	174,794	171.9	1101.2	502.8
2008	35,066,226	69,595	389,554	189,226	198.6	1110.5	539.3
2009	37,636,609	91,327	422,134	242,405	242.7	1121.3	644.0
2010	35,957,839	90,270	450,122	253,016	251.2	1251.5	703.3
2011	38,531,996	94,054	422,606	251,338	244.2	1096.8	652.1
2012	40,528,647	93,348	439,204	272,603	230.4	1083.7	672.3
2013	42,186,781	92,866	445,222	276,583	220.4	1054.8	655.3
2014	43,283,495	92,574	456,287	291,375	214.1	1053.8	672.8
2015	44,109,666	85,667	406,254	277,642	194.4	920.7	629.0
2016	43,989,831	84,172	393,925	273,289	191.5	895.0	621.1
2017	47,379,104	79,801	344,432	278,158	168.7	726.6	586.5
2018	49,067,571	79,605	334,760	293,849	162.4	682.2	598.4
2019	50,887,808	77,965	322,974	295,911	153.4	634.6	580.9
Mean	41,798,411	83,919	400,796	259,245	203.4	979.4	619.8

a Insured subjects registered with a family physician.

b Because the total number of cases and insured persons is included in each year, data is equivalent to age- and sex-adjusted incidences.

**Fig. 1:**
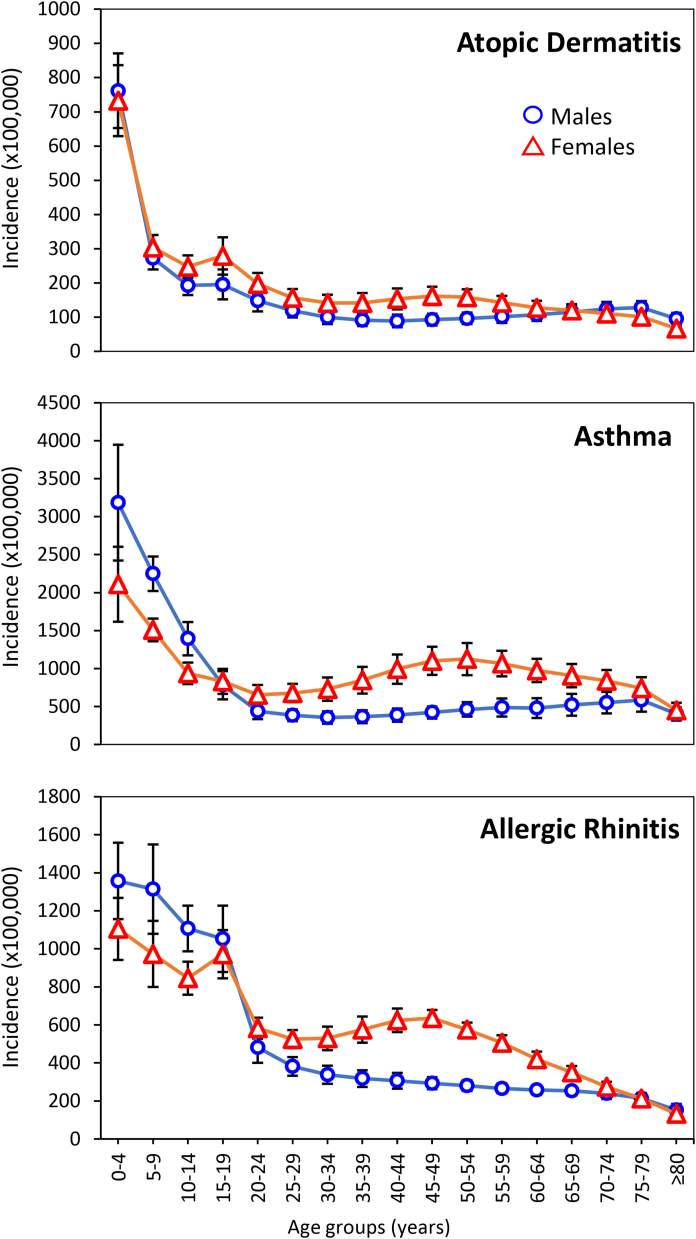
Nationwide incidence rates of atopic dermatitis, asthma and allergic rhinitis by sex and age group. Symbols represent the 13 years (2007–2019) averages and standard deviation of annual incidence calculated from registries of new cases occurring in an insured population ranging from ∼34.8 million (2007) to ∼50.9 million subjects (2019).

In males, children 0–4 years old had the highest incidence rates of the 3 atopic diseases (AD, asthma, and AR), followed by a sharp decline until adolescence for AD, a less notable drop for asthma, and even a lower decrease for AR (Fig. 1). At 20–24 years old, incidences of AD, asthma and AR represented 19.5%, 13.8% and 35.4% of the incidence at 0–4 years old, respectively. In general terms, after adolescence, incidence of all diseases continued declining until they reached a relative plateau in young adulthood, with a final drop at age 80 years and more.

In contrast, in females, after an initial decline of atopic diseases until adolescence there was a small increase in the AD and AR incidences in the 15-19 years-old group, and a second, more gradual increment of all incidences, peaking at 40–54 years old, mainly in asthma and AR.

These trends in the male and female incidences determined that during childhood males were more frequently affected than females, both for asthma and AR (M:F ratios 1.500, and 1.296, respectively), while AD did not show differences between both sexes (M:F ratio 0.953) (Table 2). After adolescence, the M:F ratio switched to a higher incidence in females than in males in all 3 atopic diseases (M:F ratios 0.519, 0.677, and 0.735, respectively).

**Table 2:** Annual average incidence (per 100,000 insured subjects) of allergic diseases and the male:female (M:F) ratios

Variables	Age groups	
	0–14 years	≥15 years
Atopic dermatitis		
Males	397.8 ± 15.0	112.7 ± 5.5
Females	417.2 ± 15.4	153.2 ± 6.9
Crude M:F ratio	0.953 ± 0.005	0.735 ± 0.007
Adjusted M:F ratio^a^	0.951 (0.946–0.956)	0.728 (0.724–0.732)
Asthma		
Males	2250.4 ± 106.9	448.6 ± 27.3
Females	1498.5 ± 68.6	857.4 ± 41.6
Crude M:F ratio	1.500 ± 0.006	0.519 ± 0.008
Adjusted M:F ratio^a^	1.499 (1.495–1.503)	0.521 (0.520–0.523)
Allergic rhinitis		
Males	1256.2 ± 45.9	362.2 ± 11.4
Females	969.0 ± 35.0	533.9 ± 11.1
Crude M:F ratio	1.296 ± 0.005	0.677 ± 0.009
Adjusted M:F ratio^a^	1.296 (1.292–1.300)	0.668 (0.666–0.670)

Data correspond to mean ± standard error of 13 years (2007–2019) or to adjusted M:F ratio (95% confidence intervals), from a population size ranging from ∼34.8 (year 2007) to ∼50.9 (year 2019) million subjects.

a Age-adjusted by Poisson regression.

**Fig. 2:**
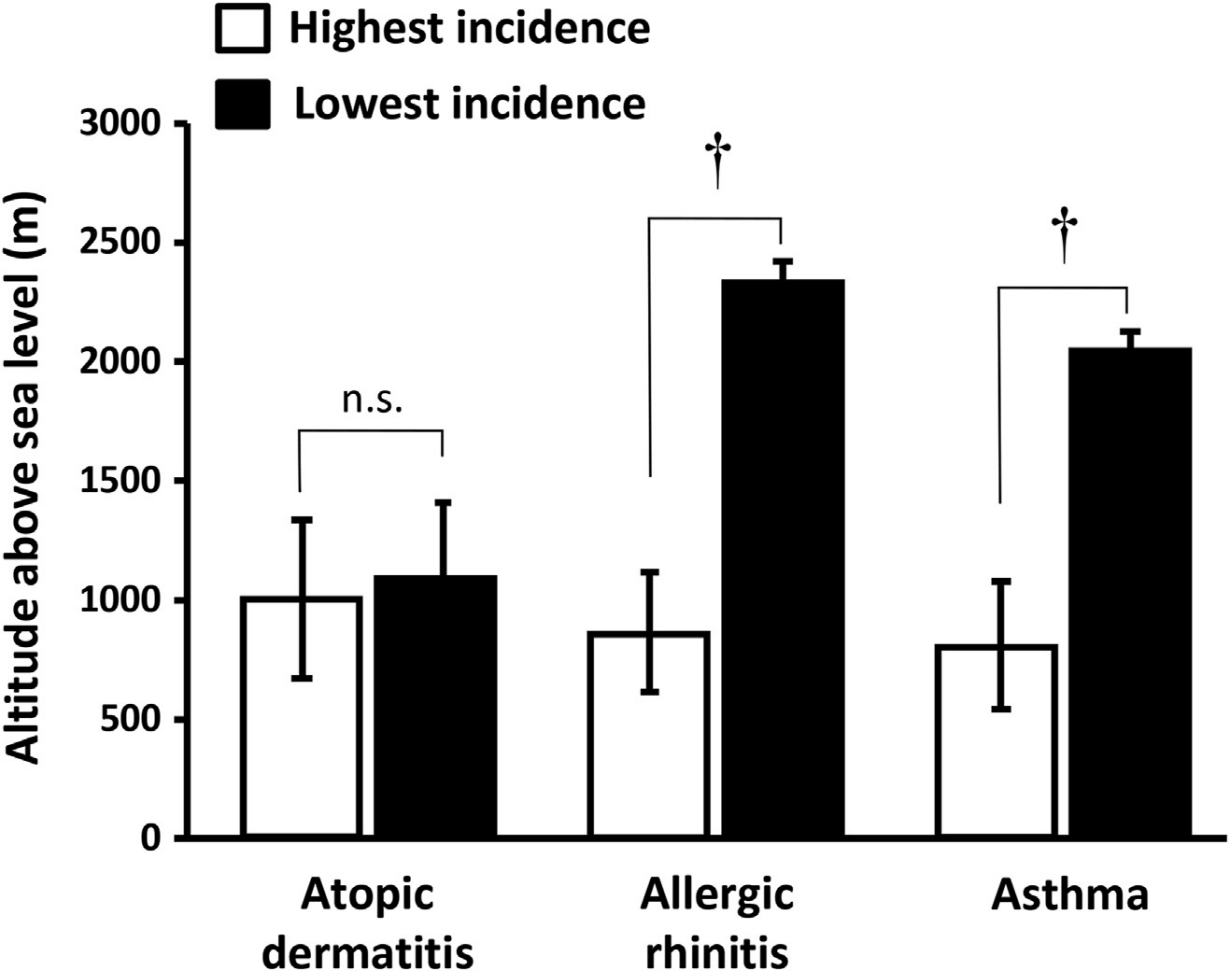
Altitude above sea level of counties with the highest and lowest incidences (evaluated as the average annual incidences 2007–2019) of atopic dermatitis, asthma, and allergic rhinitis. These counties were selected from 365 Mexican counties with >10,000 insured people. Data correspond to the mean ± standard error of the mean. †p < 0.001, n. s. = non statistically significant (nonpaired Student's *t*-test).

Fig. 2 shows altitude above sea level of the 10n counties with the highest incidences (evaluated as the average annual incidences 2007–2019) of asthma, AR, and AD, as well as the 10 counties with the lowest incidences of these diseases. As can be observed in this figure, there was a clear inverse relationship between altitude and incidences of asthma and AR, but not between altitude and AD incidence (see also Supplementary Table S1).

**Fig. 3:**
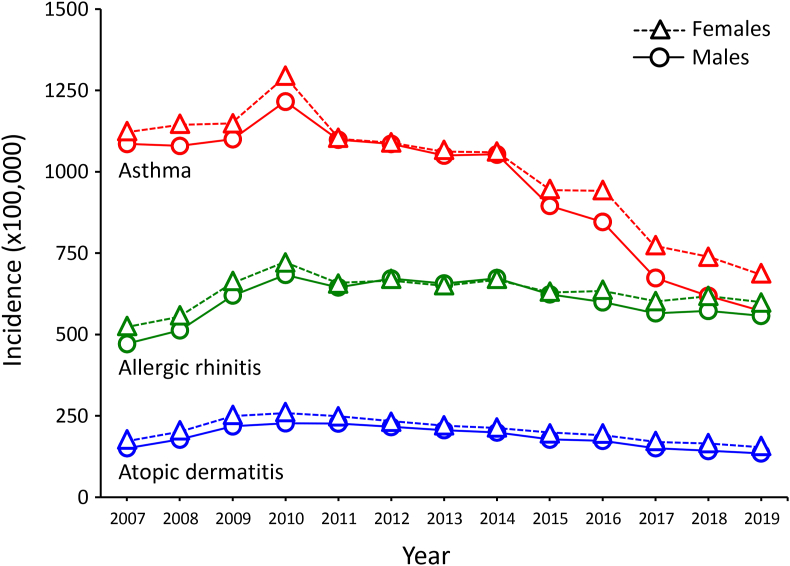
Time trends in nationwide annual incidence rates of atopic dermatitis, asthma, and allergic rhinitis. Data correspond to the cumulative number of all new cases diagnosed each year by family physicians among 34.8 million (year 2007) to 50.9 million (year 2019) insured subjects.

The time trend analysis of the 13 years studied (2007–2019) showed that the 3 atopic diseases had a descending trend from 2011 onward (Fig. 3 and Table 1). In agreement with this global tendency, when broken down by age group it was evident that annual incidences in almost all age groups peaked around 2009–2011, followed by a downward trend (Supplementary Fig. S1-S3). This latter decreasing trend was quite evident for AD (Supplementary Fig. S1) and asthma (Supplementary Fig. S2), which in 2019 reached a final drop of ∼50% and ∼40% of their maximal incidences, respectively, while for AR (Supplementary Fig. S3) it was much less notorious (∼20% drop), and even in some age-groups (eg, 5-9- and 10-14-years old groups) the increasing trend was maintained throughout the studied period. In all cases, these time trends showed a similar behavior for male and female patients.

## Discussion

In the present study, we describe the lifetime incidence patterns of the 3 major diseases involved in the atopic march in a very large population. Our results showed that these diseases had their greatest annual incidences within the first 4 years of age, with a subsequent decline thereafter, mainly in males.

Many studies have analyzed the frequency of AD, AR, and asthma according to age, yielding contrasting results. Czarnowicki et al.^23^ illustrated the atopic march with an initial peak of AD rate in the 1–3 years age group, followed by a peak of asthma at 4–6 years of age, and by a plateau of AR from 10 years of age onward. In turn, Hill and Spergel.^24^ opined that AD and asthma are diagnosed more frequently at 0–5 years of age, and at 12–17 years of age, respectively, while AR reaches a plateau from 24 years of age onward. Gough et al.^25^ showed that AD was 5 times more frequent at an age of 12 years than at 26 years. In a meta-analysis of 7 population-based cohorts addressing AD prevalence until age 26 years,^26^ the prevalence patterns in childhood and adolescence differed among the studies, being the work made in Iceland by Finnbogadottir et al.^27^ the study showing a prevalence trend that resembles our findings, ie, with the highest peak in early life and a sharp decline thereafter. Although AR is generally thought to be developed in late childhood, Eriksson et al.^28^ found that its highest prevalence occurred in subjects between 30 and 40 years of age, with a gradual decline thereafter. Our results followed at some extent this pattern, but only in females, while males had a decreasing trend of AR incidence throughout life.

The use of incidence in our study might partially explain the contrasting results with respect to those studies using prevalence, but a complex array of other factors could be also involved, such as ethnicity, environmental pollution, dietary habits, sedentary lifestyle, antibiotics use, exposure to chemicals, marginalization, and geographical conditions, among others.

With respect to incidences by sex, at least for AR and asthma our results resemble the well-known pattern in which incidence is higher among boys during childhood, but changes in adolescence, and then females become more affected than males and continue so during the rest of the life.^29^^,^^30^ We found that AD, by contrast, did not present the increased frequency in males during childhood (Table 2). This contrasting behavior of AD with respect to asthma and AR has also been documented by some cohort studies. For example, Gough et al.^25^ showed an increased prevalence of AD in females during childhood and up to 20 years of age, while asthma and AR displayed the well-known male predominance during this time period. Likewise, Burr et al.^31^ found an increased AD prevalence in females from age 7–23 years, as compared with males, and the cohort follow-up by Ziyab et al.^32^ showed that, like our results, there was a similar prevalence of AD in both sexes up to 10 years of age, with a clear female predominance from 10 to 18 years. Mohrenschlager et al.^33^ found a slight female predominance in the prevalence of AD among children aged 5–7 years, which was explained by a higher skin surface pH and lower stratum corneum hydration in girls, as compared with boys. Although the higher female frequency of atopic disease in adulthood is usually considered to be related to changes in sexual hormones, this claim is mostly based on *in vitro* and animal model studies, and it has not been proved conclusively in the clinical setting. In this context, a recent ecological study found that the M:F ratio in asthma incidence during adulthood did not parallel hormonal changes, and that factors such as population density and acute respiratory tract infections, among others, might also be involved.^34^ The lack of a major role of hormonal influence is clearly observed in Fig. 1, where inception of the atopic disease continues to be higher in women, even if they are in post-menopausal age-groups.

A worrisome epidemic increase in the prevalence of asthma, AR and AD was observed worldwide throughout the second half of the 1900s century.^35^, ^36^, ^37^ However, this rising trend became much less pronounced (or even changed to a descending trend in the case of AR) in the last 20 years.^1^^,^^36^^,^^38^ In our analysis of 13 years it was evident that after reaching a peak around 2009–2011, the annual incidence of asthma and AD began to decrease in all age groups, while yearly incidence of AR seemed to maintain a flat trend or even to increase in some age groups. In the last few years, a number of reports from several countries have evaluated the time trends of major atopic diseases by analyzing at least 3 different time-points.^36^^,^^39^, ^40^, ^41^, ^42^, ^43^, ^44^, ^45^, ^46^, ^47^, ^48^, ^49^, ^50^, ^51^, ^52^, ^53^, ^54^ As can be seen in the Supplementary Fig. 4, in general terms their results are in agreement with ours in the sense that the frequency of these diseases appear to be decreasing in recent years. The cause of the decreasing trend of the major atopic diseases, particularly asthma, is largely unknown. One of the most widely accepted explanation of the epidemic rise of allergic diseases is the hygiene hypothesis, which postulates that the lower exposure to microorganisms or their products conveys an immunological bias toward allergy.^55^ It has been proposed that *Streptococcus pyogenes*, the causal agent of scarlet fever, might be involved in the hygiene hypothesis due to the strong temporal, seasonal and geographical inverse association of scarlet fever and asthma.^56^ In this context, an unexpected rise in the incidence of scarlet fever has been well documented in several regions of the world from 2011 onward.^57^, ^58^, ^59^, ^60^, ^61^ Such an increase in the incidence of scarlet fever probably implies that the rate of asymptomatic nasopharyngeal carriage of *Streptococcus pyogenes* in the general population was also increased. Thus, although we have no evidence that the incidence of scarlet fever has increased in Mexico, it may be possible that a higher frequency of *Streptococcus pyogenes* carriage explains the decreasing trend of asthma (and AD) from 2011 onward. An alternative explanation is based in the strong inverse relationship between neural tube defects and asthma, already demonstrated in Mexico and the United States.^62^ By analyzing the official health data on annual incidences of asthma and spina bifida in Mexico,^63^ we could corroborate that both diseases still maintained a strong inverse temporal association during the same time-period used in the present study (Supplementary Fig. S5). A possible explanation of this association is that folic acid, which is a well-known preventer of neural tube defects, might be simultaneously promoting the development of asthma and/or other atopic diseases via epigenetic mechanisms such as histone methylation.^64^

Interestingly, AD exhibited some differences with respect to asthma and AR. For example, AD incidence did not show the male predominance during childhood, as occurred with asthma and AR (Table 2), nor have the same ecological association with altitude as that seen for asthma and AR (Fig. 2). With respect to the latter association, a more detailed analysis of our database indeed showed that AR incidence, but not AD incidence, progressively decreased above ∼1500 m altitude (data not shown), similar to the behavior of asthma incidence already reported by us.^18^ This association between atopic diseases and altitude deserved a more in-depth analysis, and we will be reporting it in a forthcoming publication. Our results suggest that although the 3 diseases share an atopic background involving a type-2 inflammatory immune response, AD may have different underlying pathogenic mechanisms, a concept that needs further investigation. Likewise, these differences reinforce the notion that the tracheobronchial tree and the nose are part of a “common airway” affected by the same sensitization process manifested by asthma and AR, respectively.

Finally, it was clear that incidences of the studied atopic diseases progressively declined after 80 years of age, coinciding with the already reported low prevalence of allergic disease in this age-group.^65^^,^^66^ Thus, the low rates of AD, AR and asthma in elderly people might be related to the well-known decrement of cellular and humoral immune responses with aging,^67^ although a survival bias cannot be discarded.

Strengths of our study were that: a) the extensive period analyzed (13 years) allowed us to confidently depict recent time trends of the major atopic diseases; b) a very large population ranging from ∼34.8 (2007) to ∼50.9 (2019) million insured subjects was evaluated, comprising ∼744,000 new cases of AD, AR or asthma diagnosed each year; c) we used incidence rates instead of prevalence, so a better picture of disease inception was obtained; and d) diagnoses were established by family physicians, avoiding the usual biases and large overestimation from questionnaires or surveys.^68^ Conversely. because all patients were seen at first level medical units, which lack allergic skin tests or other procedures, a potential limitation of our study was that allergenic sensitization could not be confirmed. On the other hand, although it is possible that some degree of uncertainty exists in the accuracy of clinical diagnoses (specially the diagnosis of asthma in young children) or ICD codes capture, we consider that these would be minimal because in our institution it is mandatory for all family physicians to diagnose these atopic diseases according to established clinical practice guidelines, and encode technicians are trained personnel specialized in this task.

## Conclusions

Herein we described the epidemiological profile of 3 major diseases involved in the atopic march (AD, AR, and asthma) during the life span in a very large population from a developing country. Our results showed that these diseases had their highest incidence in infancy and corroborated that, at least for asthma and AR, their incidence during childhood is higher in males, as compared with females, with a shift to a female predominance in adulthood. Additionally, we found that annual incidences of AR and asthma started to decline in most age-groups from ∼2009–2011 onward, and that the counties with the highest incidence of AR and asthma were those located at lower geographical altitude. Because AD exhibited a somewhat contrasting pattern with respect to AR and asthma, ie, a different age and sex distribution and a lack of association with geographical altitude, it is possible that its underlying mechanisms determining the atopic background differ from those of AR and asthma.

## Funding source

None.

## Authors’ contributions

MBA and MHV designed the study, performed the statistical analysis, interpreted the results and wrote the manuscript draft. ISMR and RARP contributed to data collection and critically reviewed the manuscript for important intellectual contribution. All authors read and approved the final manuscript.

## Clinical Trial registration

Not applicable.

## Ethics approval

Because this was a retrospective analysis of a de-identified institutional database, it was deemed unnecessary obtaining the approval from an ethics and scientific review board.

## Authors’ consent for publication

All authors have read through and approved the publication of the manuscript.

## Availability of data and materials

The original data are not publicly available because it is a very large institutional database with privacy restrictions.

## Potential competing interests

The authors report no competing interests.
