# Supplementary material for: Trends (2007–2019) of major atopic diseases throughout the life span in a large Mexican population
Source: World Allergy Organ J. 2023 Jan 9;16(1):100732. doi: 10.1016/j.waojou.2022.100732 (PMC9841056; doi:10.1016/j.waojou.2022.100732)

Supplementary material

Trends (2007-2019) of major atopic diseases throughout the life span in a large Mexican population

Table S1. Mexican counties with the highest and lowest incidences of asthma, allergic rhinitis, and atopic dermatitis

Figure S1. Time trends in incidence of atopic dermatitis by age-group and year.

Figure S2. Time trends in incidence of asthma by age-group and year.

Figure S3. Time trends in incidence of allergic rhinitis by age-group and year.

Figure S4. Time trends of major atopic diseases reported in recent published studies.

Figure S5. Nationwide temporal association of asthma and spina bifida incidences.

Table S1. Mexican counties with the highest and lowest incidences of asthma, allergic rhinitis, and atopic dermatitis

Asthma					Allergic rhinitis					Atopic dermatitis				
County, State	Altitude (m)	Insured population	Cases	Incidence (x100,000)	County, State	Altitude (m)	Insured population	Cases	Incidence (x100,000)	County, State	Altitude (m)	Insured population	Cases	Incidence (x100,000)
					Highest incidence									
Tlajomulco, Jalisco	1561	46498	2223	4164.3	Lerdo de Tejada, Veracruz	10	14488	514	3470.5	San Pablo del Monte, Tlaxcala	2340	13397	96	726.3
Villa Corona, Jalisco	1373	11811	393	3365.8	La Antigua, Veracruz	19	42421	1361	3182.8	Úrsulo Galván, Veracruz	21	12140	84	679.7
Atizapán, México	2286	82423	1975	2437	Tlajomulco, Jalisco	1561	46498	1443	2503.4	Acanceh, Yucatán	10	16339	94	654.8
Tamazula, Jalisco	1166	21577	520	2432.1	Cosamaloapan, Veracruz	11	54336	980	1849.3	Azcapotzalco, Ciudad de México	2249	200335	1256	646.4
Ángel R. Cabada, Veracruz	47	12130	289	2429	Saucillo, Chihuahua	1212	18452	325	1739.8	Tlaxcala, Tlaxcala	2282	57675	337	596.3
Motul, Yucatán	2	27964	652	2420.6	Atizapán, México	2286	82423	1421	1714.4	Huatulco, Oaxaca	115	20291	111	570.5
Tala, Jalisco	1352	55354	1296	2406.6	Salina Cruz, Oaxaca	21	28034	440	1710.4	Cunduacán, Tabasco	8	10876	52	467
Autlán, Jalisco	925	33610	746	2269.5	Santiago Ixcuintla, Nayarit	18	34516	568	1638.8	Xalapa, Veracruz	1403	69161	318	462.5
Lerdo de Tejada, Veracruz	10	14488	322	2259.3	Cuernavaca, Morelos	1526	248090	3970	1573.8	Izamal, Yucatán	18	18916	78	459.2
Tuxpan, Nayarit	9	14556	323	2255.8	Xalapa, Veracruz	1403	69161	1030	1545.8	Tlajomulco, Jalisco	1561	46498	226	446.7
Mean ± s.e.m.	873.1 ±257.6			2644.0 ± 197.5	Mean ± s.e.m.	806.7 ± 277.3			2092.9 ±223.7	Mean ± s.e.m.	1000.7 ± 335.8			570.94 ± 33.3
					Lowest incidence									
Corregidora, Querétaro	1858	69684	51	72.6	Romita, Guanajuato	1759	12112	16	132.1	Francisco I. Madero, Coahuila	1108	45606	30	68.3
Tenango del Valle, México	2667	39964	29	70.7	San Luis de la Paz, Guanajuato	2029	29715	24	83.7	San Pedro, Coahuila	1096	46502	22	50.2
Jilotzingo, México	2774	10272	7	64.8	Silao, Guanajuato	1800	87659	101	114.6	Tecomán, Colima	32	44537	27	62.7
Apetatitlán, Tlaxcala	2295	17040	10	63.7	Mixquiahuala, Hidalgo	2005	20860	13	71.3	Abasolo, Guanajuato	1705	13259	7	51.3
Cuautitlán, México	2254	238291	146	63.1	Tlaxcoapan, Hidalgo	2068	18387	17	90.7	Silao, Guanajuato	1800	87659	48	55.4
Atenco, México	2244	13947	8	60.3	Ixtlahuaca, México	2555	50408	59	117.4	Atlacomulco, México	2586	35692	20	60.3
Ixtlahuaca, México	2555	50408	26	53.8	Huejotzingo, Puebla	2296	16798	20	121.7	Ciénega de Flores, Nuevo León	403	21001	7	33
Colón, Querétaro	1984	11840	6	53.5	Tepeaca, Puebla	2235	13526	12	96.6	Tepeaca, Puebla	2235	13526	7	55.3
Ozumba, México	2307	10169	5	52.9	Pedro Escobedo, Querétaro	1940	36110	39	108.3	Reynosa, Tamaulipas	34	385916	259	67.8
Otumba, México	2389	25695	9	39.8	Tequisquiapan, Querétaro	1901	22921	30	130.3	Agua Dulce, Veracruz	11	10038	6	57.9
Mean ± s.e.m.	2332.7 ± 89.6			59.5 ± 3.1	Mean ± s.e.m.	2058.8 ± 77.0			106.7 ± 6.5	Mean ± s.e.m.	1101.0 ± 303.7			56.2 ± 3.2

Data from each county corresponds to the altitude and the average 2007-2019 of annual number of cases, insured population, and incidences.

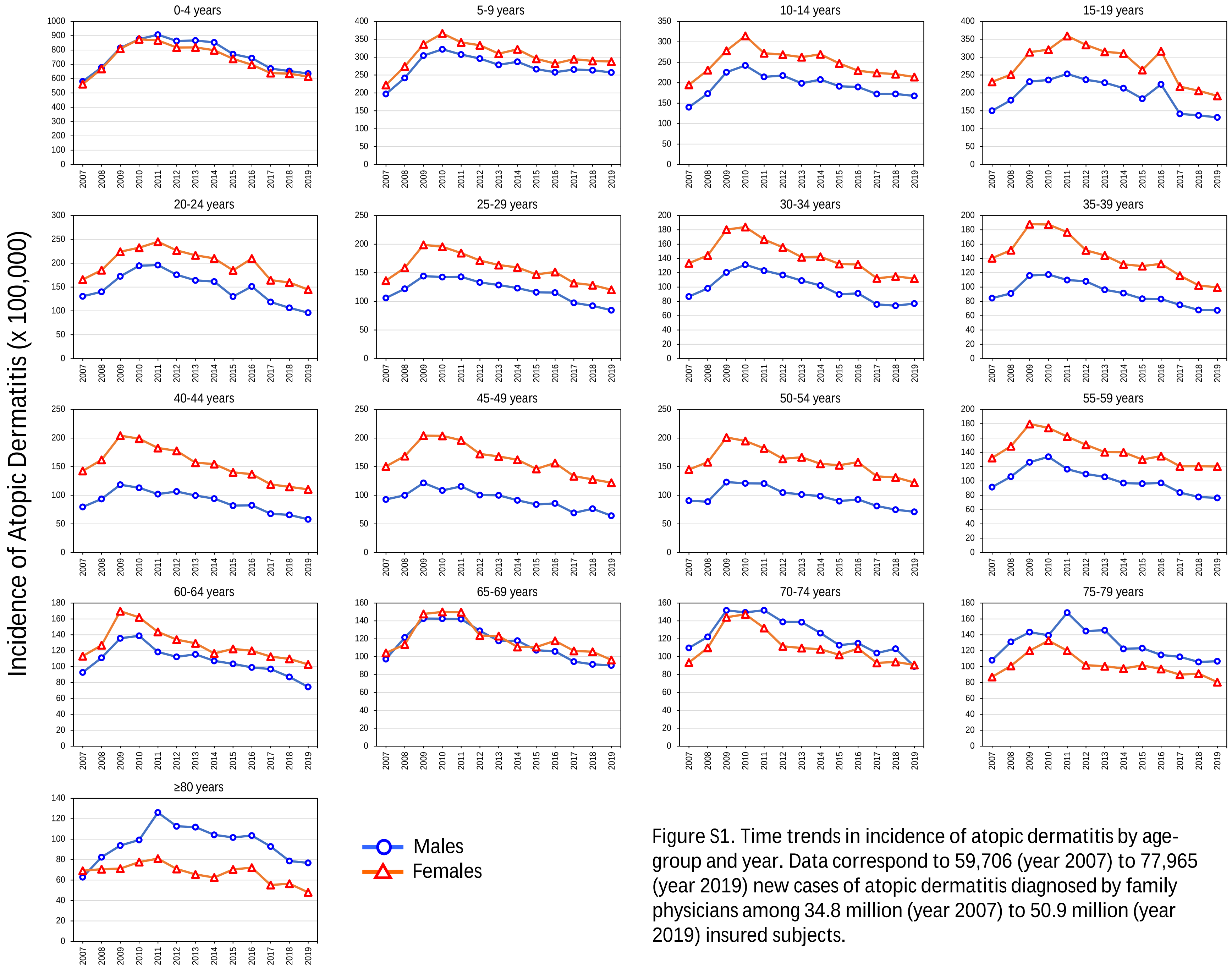

Figure S1. Time trends in incidence of atopic dermatitis by age-group and year. Data correspond to 59,706 (year 2007) to 77,965 (year 2019) new cases of atopic dermatitis diagnosed by family physicians among 34.8 million (year 2007) to 50.9 million (year 2019) insured subjects.

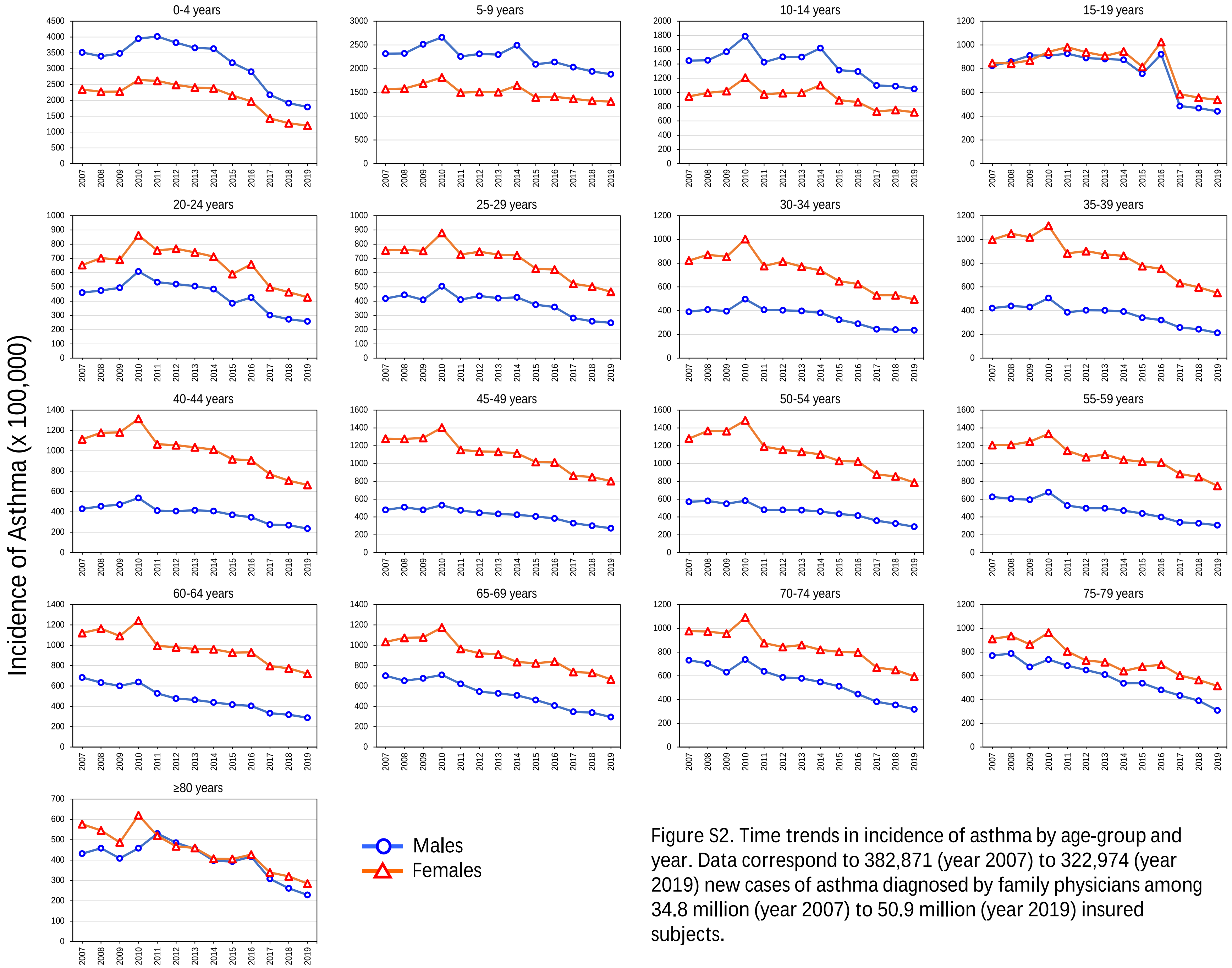

Figure S2. Time trends in incidence of asthma by age-group and year. Data correspond to 382,871 (year 2007) to 322,974 (year 2019) new cases of asthma diagnosed by family physicians among 34.8 million (year 2007) to 50.9 million (year 2019) insured subjects.

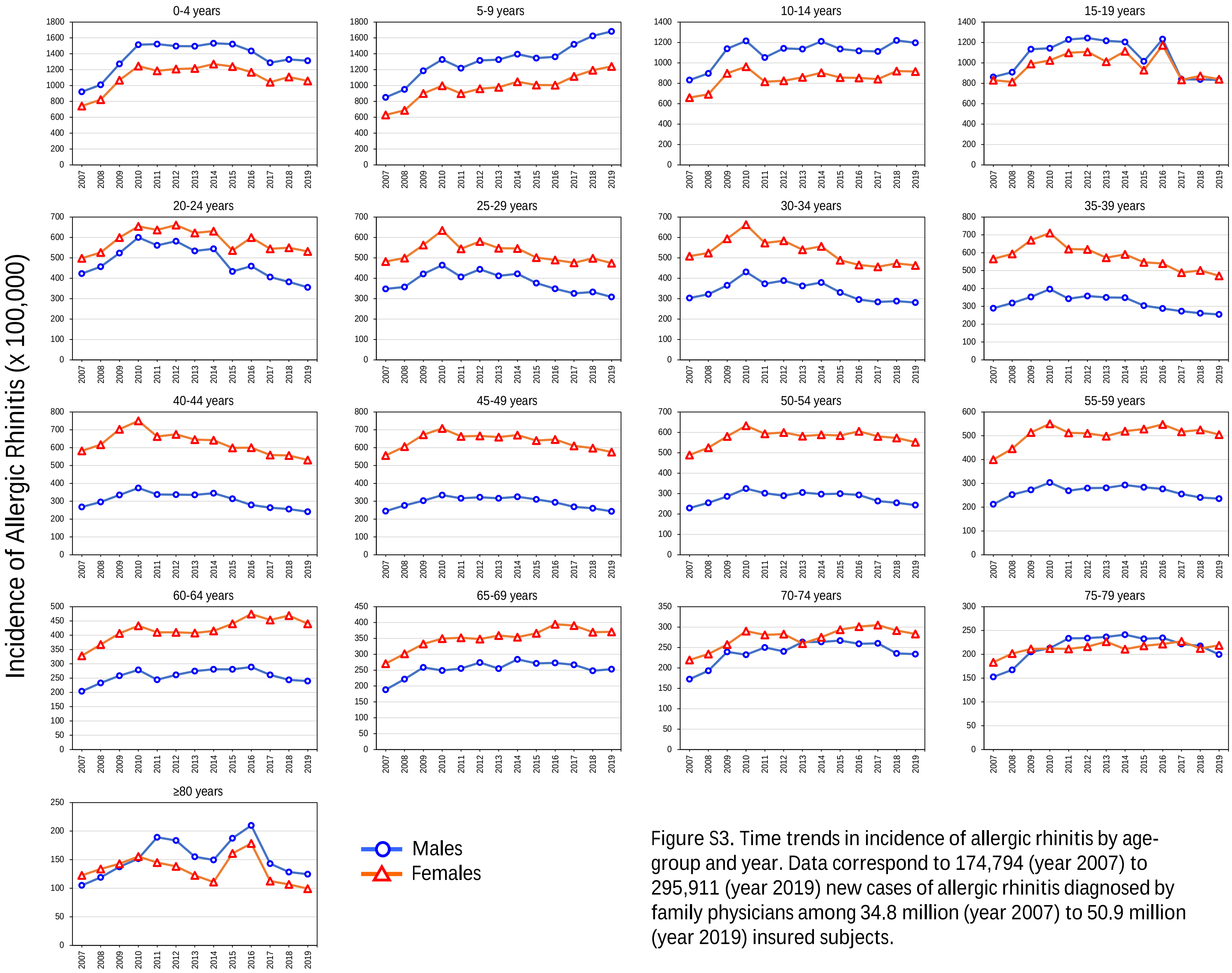

Figure S3. Time trends in incidence of allergic rhinitis by age-group and year. Data correspond to 174,794 (year 2007) to 295,911 (year 2019) new cases of allergic rhinitis diagnosed by family physicians among 34.8 million (year 2007) to 50.9 million (year 2019) insured subjects.

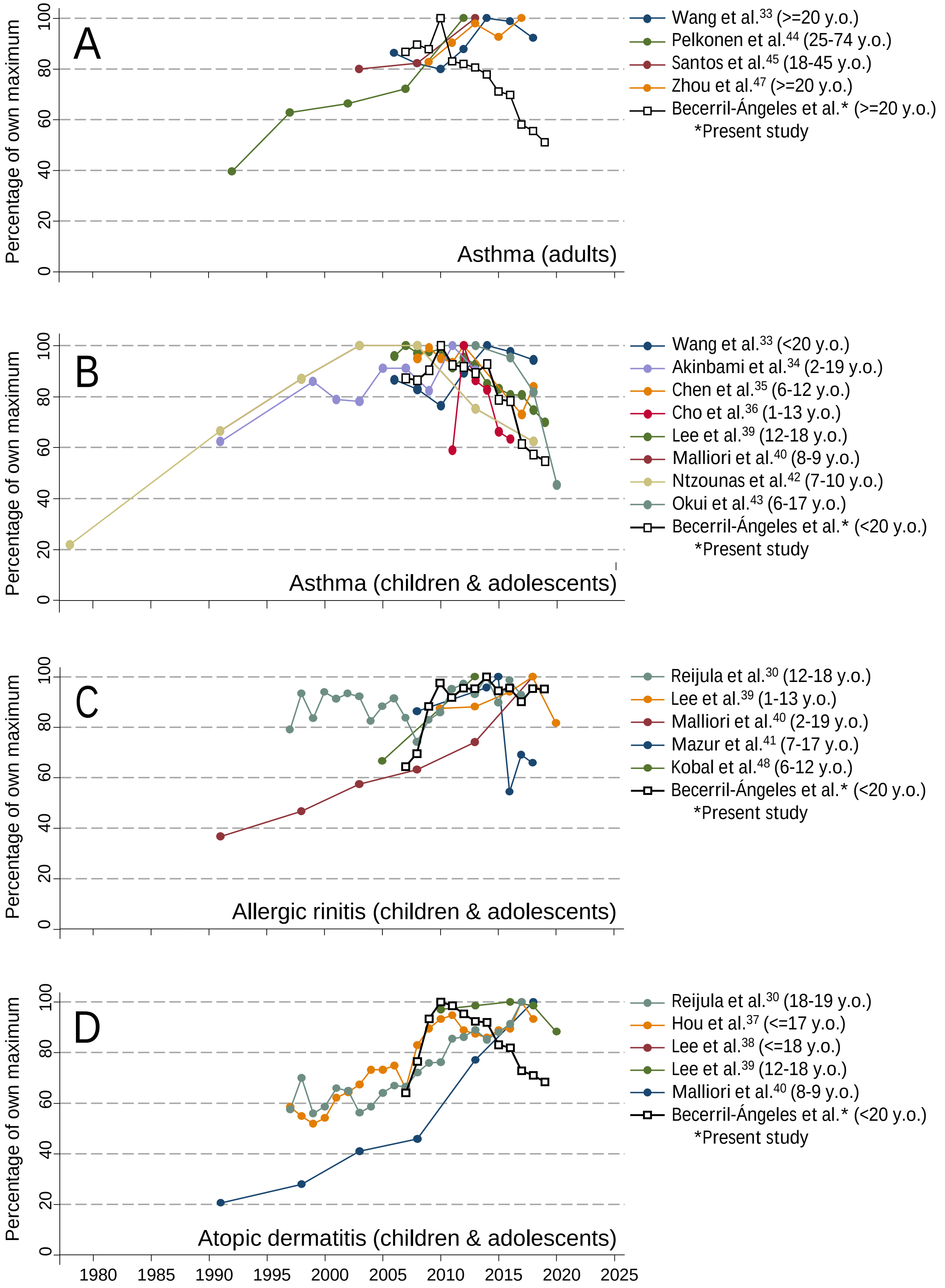

Figure S4. Time trends of major atopic diseases reported in recent published studies. Data correspond to incidences or prevalences, expressed as percentage of the maximum value achieved at any time point, of asthma (A and B), allergic rhinitis (C) and atopic dermatitis (D).

Figure S5. Nationwide temporal association (2007-2019) of asthma and spina bifida incidences per 100,000 inhabitants in Mexico.

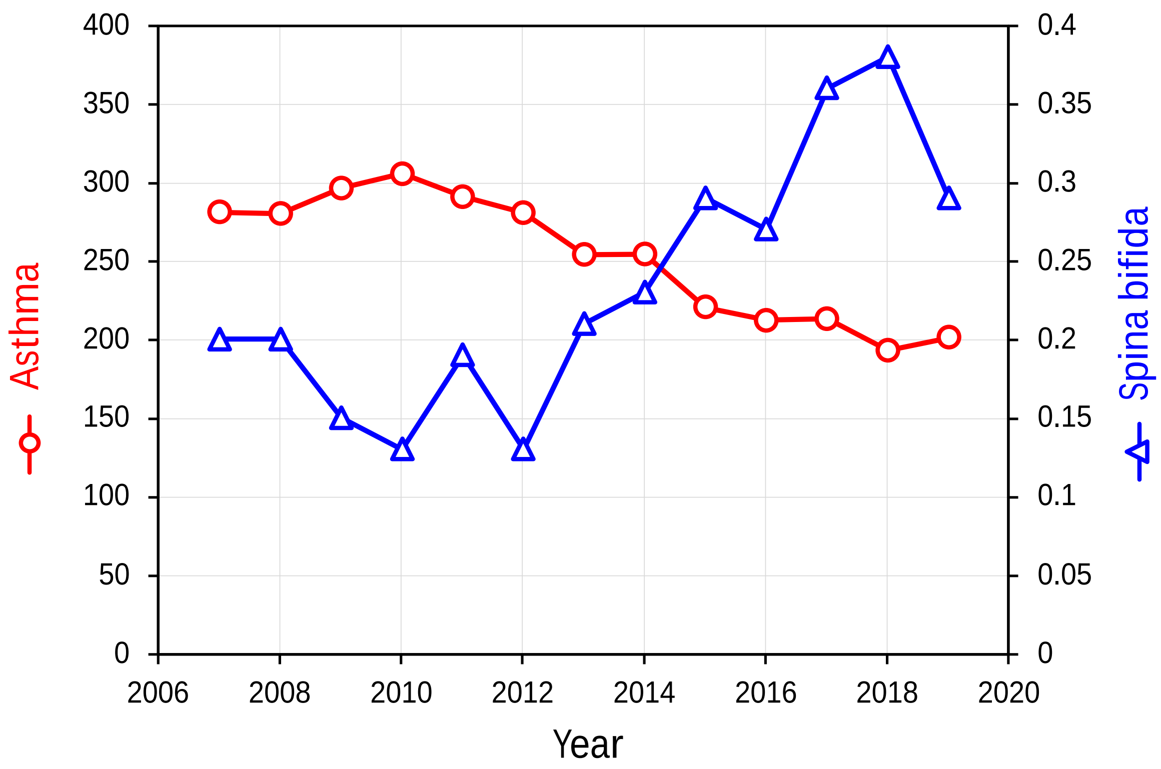

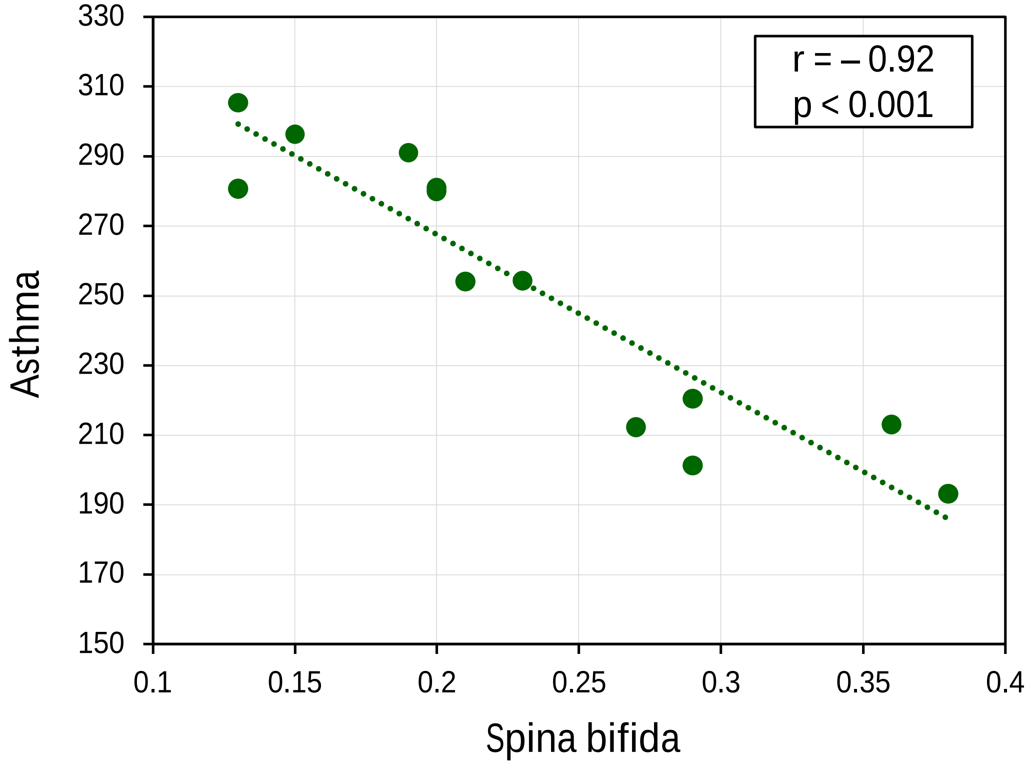

Supplement: Multimedia component 1 [file mmc1.pdf]
